# A Comprehensive Review on the Viscoelastic Parameters Used for Engineering Materials, Including Soft Materials, and the Relationships between Different Damping Parameters

**DOI:** 10.3390/s24186137

**Published:** 2024-09-23

**Authors:** Hasan Koruk, Srinath Rajagopal

**Affiliations:** Ultrasound and Underwater Acoustics Group, Department of Medical, Marine and Nuclear, National Physical Laboratory, Teddington, Middlesex TW11 0LW, UK; srinath.rajagopal@npl.co.uk

**Keywords:** damping, complex modulus, loss factor, characterisation, soft material, ultrasound, viscoelastic properties, viscosity, dynamic indentation, rheometry

## Abstract

Although the physical properties of a structure, such as stiffness, can be determined using some statical tests, the identification of damping parameters requires a dynamic test. In general, both theoretical prediction and experimental identification of damping are quite difficult. There are many different techniques available for damping identification, and each method gives a different damping parameter. The dynamic indentation method, rheometry, atomic force microscopy, and resonant vibration tests are commonly used to identify the damping of materials, including soft materials. While the viscous damping ratio, loss factor, complex modulus, and viscosity are quite common to describe the damping of materials, there are also other parameters, such as the specific damping capacity, loss angle, half-power bandwidth, and logarithmic decrement, to describe the damping of various materials. Often, one of these parameters is measured, and the measured parameter needs to be converted into another damping parameter for comparison purposes. In this review, the theoretical derivations of different parameters for the description and quantification of damping and their relationships are presented. The expressions for both high damping and low damping are included and evaluated. This study is considered as the first comprehensive review article presenting the theoretical derivations of a large number of damping parameters and the relationships among many damping parameters, with a quantitative evaluation of accurate and approximate formulas. This paper could be a primary resource for damping research and teaching.

## 1. Introduction

Soft materials exhibit both viscous (damping) and elastic (stiffness) characteristics [1,2,3,4]. The quantification of the viscoelastic properties of soft materials is essential in numerous science and engineering applications [5,6,7,8,9,10,11,12]. Furthermore, next to elasticity, damping (or viscosity) could be an additional relevant diagnostic biomarker, and viscosity could enhance the current diagnosis in quantitative elastography [13,14,15,16,17,18,19,20,21,22]. Briefly, damping is the removal of energy from a system, and the energy can be either dissipated within the system or transmitted away by radiation [23]. It should be remembered that material damping is energy dissipation due to deformation in a medium, and radiation damping is the energy transfer to a surrounding medium [23,24]. In addition, the energy in a system can be dissipated, for example, via the friction between different parts in the system and air resistance [25]. The properties of a structure such as mass and stiffness can be determined by performing some static tests. However, identifying the damping of a structure or system requires a dynamic test [26]. In general, both theoretical modelling and experimental identification of damping are quite difficult [24,27,28,29]. There are many research papers on determining the damping of materials, including biomaterials (e.g., [30,31,32,33,34,35,36,37]). The literature survey shows that there are different techniques for the identification of damping (e.g., dynamic indentation method, logarithmic decrement method, and rheometry), and each method gives a different damping parameter, such as the loss factor, loss modulus, and viscous damping ratio [23,26,38,39,40,41,42,43]. The identification of the damping of conventional materials (such as ceramics and metals) is quite straightforward, and the loss factor or viscous damping ratio is commonly used to quantify their damping [44]. On the other hand, the identification of the damping of soft materials (e.g., agar, gelatine, and collagen phantoms and tissue) is challenging, and different damping parameters such as the loss modulus, loss angle, viscous damping ratio, or viscosity are used to describe their damping [30,34,36,45,46,47].

Regarding the identification of the damping of materials, Nayar et al. [30] used the dynamic indentation method to determine the storage and loss moduli of some samples of agar, which is a representative material for biological tissues. Similarly, using the dynamic indentation method, Vriend et al. [48] determined the viscoelastic properties of some elastomeric materials, and Boyer et al. [49] assessed the stiffness and damping of skin. Dakhil et al. [31] identified the storage and loss moduli of cells using a rheometer. Peng et al. [32] determined the dilute solution viscosities of some cellulose nanocrystal dispersions using a capillary viscometer. Cartagena-Rivera et al. [50] identified the loss and storage moduli of live cells using an atomic force microscope. Shahmansouri et al. [51] identified the loss factor of the human aorta using a biaxial tensile test and hysteresis loop. Vogel and Pioletti [52] determined the specific damping capacity of the bovine nucleus pulposus using a dynamic compression test and hysteresis loop. Wang et al. [33] identified the viscous damping ratios of some beam-like hydrogel samples using resonant vibration tests. Esmaeel et al. [36] determined the viscous damping coefficient of tissue-mimicking silicon rubber samples by calculating the dissipated energy per cycle of harmonic motion by the material and the maximum stored energy in the sample using the displacement–force curve. Rosicka et al. [53] identified the biomechanical and viscoelastic properties of skin, including the logarithmic decrement values. Based on the mathematical models for the dynamic response of a microbubble located at the soft medium interface [54,55,56], Bezer et al. [34] determined the shear modulus and viscosity of a tissue-mimicking gelatine phantom by matching the experimentally measured and predicted responses of the microbubble located at the soft medium interface exposed to ultrasound. Similarly, using the mathematical models for the dynamic response of a sphere located at the soft medium interface [57,58,59], the shear modulus and viscous damping ratio of tissue-mimicking gelatine phantoms were identified by matching the experimentally measured and predicted responses of the sphere located at the soft medium interface [37,60,61].

Li et al. [62] presented the viscoelasticity imaging of biological tissues and single cells using shear wave propagation, including examples of ultrasound shear wave viscoelasticity imaging applications. Beuve et al. [63] used diffuse shear wave spectroscopy for the characterisation of the viscoelastic properties (shear modulus and viscosity) of soft tissue. Tecse et al. [64] developed and validated a method for the characterisation of the viscoelastic properties of soft tissue using ultrasound elastography. Wang et al. [65] investigated the effect of damping on ultrasound elastography algorithms. Koruk and Pouliopoulos [66] presented elasticity and viscoelasticity imaging based on the use of small particles located within the tissue and at the tissue interface while being exposed to static and dynamic external forces. Hirsch et al. [45] measured the shear moduli and loss angles of the liver and spleen using magnetic resonance elastography. Wang et al. [67] derived the shear wave speed and loss angle for depicting hepatic fibrosis and inflammation in chronic viral hepatitis using magnetic resonance elastography. The damping parameters of various materials identified using different methods in the literature are listed in Table 1. It should be noted that each method has its advantages and limitations [29,33,36,51,67,68,69].

Overall, the literature review shows that the dynamic indentation method [30], rheometry and viscometry [31,32,70], atomic force microscopy [71], hysteresis loop [36], resonant vibration tests or experimental modal analysis [33,72], and logarithmic decrement [47,53] are commonly used to identify the damping of materials, including soft materials. In addition, a bubble or sphere placed inside the soft medium or located at a soft medium interface that is exposed to external excitation, such as acoustic radiation force or magnetic force, has been recently used to identify the viscoelastic properties of soft materials [34,37,73,74,75,76,77]. The use of ultrasound elastography [17,69,78,79] and magnetic resonance elastography [80,81,82,83] for determining the mechanical properties of tissue is quite common in preclinical and clinical applications. It is seen that there are many parameters for the description and quantification of damping. The viscous damping ratio, loss factor, complex modulus (or storage and loss moduli), and viscosity are quite commonly used to describe the damping of materials. In addition, some other parameters, such as the specific damping capacity, phase lag or loss angle, half-power bandwidth, logarithmic decrement, and inverse quality factor, are used to describe the damping of various materials. Often, one of these parameters (e.g., loss factor) is measured in practical applications, and for comparison purposes, the measured damping parameter needs to be converted into some other damping parameters (e.g., viscosity). However, there is a limited number of studies that have evaluated a few different damping parameters and presented their relationships [38,84,85]. Therefore, there is a need for a comprehensive study that presents the theoretical derivations of different damping parameters and their relationships. 

This paper presents the theoretical derivations of different parameters for the description and quantification of damping and their relationships. In this paper, the expressions for both high damping (i.e., accurate formulas) and low damping (i.e., approximate formulas) are presented, and these approaches are evaluated. The structure of this paper is as follows: First, the elastic, viscous, and viscoelastic materials are defined, and then the responses of single-degree-of-freedom (SDOF) systems with a viscous damper and a complex stiffness are presented in Section 2. By exploiting the theoretical background presented in Section 2, the theoretical derivations of different damping parameters and their relationships are presented in Section 3. It should be noted that MATLAB (R2022a) software (MathWorks, Natick, MA, USA) was used to present the relationships among different parameters whenever needed. The damping parameters investigated in this paper include the specific damping capacity, loss factor, viscous damping coefficient, viscous damping ratio, phase lag (or loss angle), logarithmic decrement, half-power bandwidth, complex modulus (or loss and storage moduli), inverse quality factor, viscosity, decay ratio in the step response, and structural reverberation time. The relationships between different damping parameters are summarised in Section 4, and some sample damping identification applications of biomaterials using different sensing technologies are presented in Section 5. It is anticipated that many researchers conducting research on damping, from very soft materials to very stiff conventional engineering materials used in different fields, will refer to this study. In addition, the material presented in this study can be exploited for teaching about damping or viscoelasticity in various branches. 

## 2. Theoretical Background

In the following sections, the elastic, viscous, and viscoelastic materials are first defined, and then the responses of SDOF systems with a viscous damper and a complex stiffness are presented. It is worth remembering that by using the theoretical background presented in this section, different damping parameters are derived, and their relationships are presented in Section 3.

### 2.1. Elastic, Viscous, and Viscoelastic Materials

Materials are mostly assumed to behave according to Hooke’s linear elasticity theory under small deformations. In other words, it is assumed that there is a linear relationship between the stress and strain, given by:

(1) σ=Eε

where σ, E, and ε are the stress, Young’s modulus, and strain, respectively. It should be noted that the same relation can be written between the shear stress and strain as τ=Gγ, where τ, G, and γ are the shear stress, shear modulus, and shear strain, respectively. In this article, the expressions are written mostly using the normal strain, normal stress, and Young’s modulus. However, it should be kept in mind that similar expressions can be written using the shear strain, shear stress, and shear modulus. The materials described in Equation (1) are called elastic materials. For a so-called purely elastic material, all the energy stored in the sample during loading is returned when the load is removed. Engineering materials such as aluminium and steel can be conveniently assumed as elastic materials. 

Opposite to an elastic material, a so-called purely viscous material does not store energy. For a purely viscous material, there is no elastic component, and all the energy is dissipated as pure damping once the load is removed. For these materials, the stress is proportional to the strain rate, given by:

(2) $$\sigma =\mu \frac{d\varepsilon }{dt}$$

where μ is the viscosity, and $\dot{\varepsilon }=\frac{d\varepsilon }{dt}$ is known as the strain rate. Liquidus materials such as glycerine, oil, and honey can be considered as viscous materials. 

The so-called viscoelastic materials show both elastic and viscous behaviours; therefore, they exhibit time-dependent strain [86,87]. For viscoelastic materials, some of the energy stored in the system can be recovered upon the removal of the load, and the remaining energy is dissipated in the form of heat. There are different mathematical models, such as the Kelvin–Voigt, Maxwell, and standard linear solid models, for the viscoelastic materials where springs and dampers are arranged in series and/or parallel to determine their stress and strain relationships [86,88,89,90,91]. For example, for the Kelvin–Voigt model which is represented by a purely viscous damper and purely elastic spring connected in parallel, the stress, strain, and strain rate with respect to time are governed by [92]:

(3) $$\sigma \left(t\right)=E\varepsilon \left(t\right)+\mu \frac{d\varepsilon \left(t\right)}{dt}$$

Tissue-mimicking materials such as hydrogels and biological structures such as tissue and skin show viscoelastic behaviour. 

**Figure 1:**
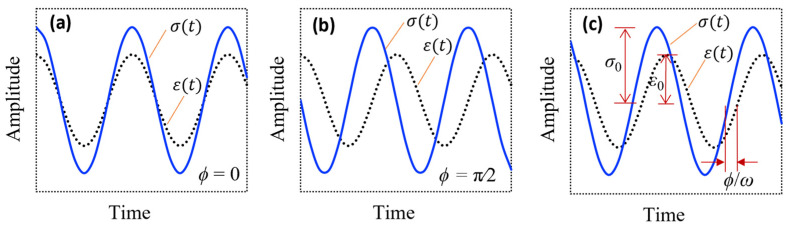
The cyclic stress–strain versus time plots for classical elastic (**a**), viscous (**b**), and viscoelastic (**c**) materials.

The cyclic stress–strain versus time for classical elastic, viscous, and viscoelastic materials are shown in Figure 1. The stress and strain curves for elastic materials move completely in phase, as seen in Figure 1a, while there is a π/2 radian or 90° phase difference between the stress and strain for viscous materials, as seen in Figure 1b [93]. On the other hand, with the cyclic stress at frequency ω, there is a phase ϕ between the stress and strain for viscoelastic materials, where ϕ is between 0 and π/2 (Figure 1c). It is noted that $\varepsilon_{0}$ and $\sigma_{0}$ show the strain and stress amplitudes, respectively. The term ϕ is also called the phase shift or loss angle. It should be noted that the loss angle is a measure of a material’s damping. 

Various formulations for the response of an SDOF system are given in Section 2.2, Section 2.3 and Section 2.4. By exploiting the theoretical background presented in Section 2.2, Section 2.3 and Section 2.4, the theoretical derivations of different damping parameters and their relationships are presented in Section 3. 

### 2.2. Viscously Damped SDOF System Exposed to Harmonic Excitation

#### 2.2.1. Steady-State Response of a Spring–Damper System

**Figure 2:**
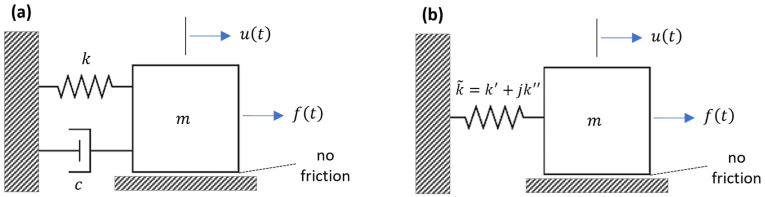
A viscously damped SDOF system (**a**) and an SDOF system with a complex stiffness (**b**).

The equation of motion for a viscously damped SDOF system with damping coefficient c and spring coefficient k without any inertia (i.e., m=0) exposed to a harmonic excitation $f\left(t\right)=F_{0}sin\left(\omega t\right),$ shown in Figure 2a, can be written as follows:

(4) $$c\dot{u}+ku=F_{0}sin\left(\omega t\right)$$

where $F_{0}$ is the amplitude of the applied force, u and $\dot{u}$ show the displacement and velocity, respectively, ω=2πf is the angular or circular frequency in rad/s, and f is the linear frequency in 1/s or Hz. The steady-state solution for this system can be written as follows [38]:

(5) $$u(t)=Bsin\left(\omega t-\phi \right)$$

where B is the amplitude of the steady-state response, and ϕ is the phase angle by which the response lags the excitation given by:

(6) $$B=\frac{F_{0}}{k\sqrt{1+{\left(\frac{c\omega }{k}\right)}^{2}}}=\frac{F_{0}}{k\sqrt{1+{tan}^{2}\phi }}$$

(7) $$\phi ={tan}^{-1}\left(\frac{c\omega }{k}\right)$$

It is seen that the phase angle is a function of the material properties (i.e., c and k) and the frequency (ω) for a viscously damped system.

#### 2.2.2. Free Vibrations of a Mass–Spring–Damper System

The equation of motion for a viscously damped SDOF system with damping coefficient c, mass m, and spring coefficient k without any external force (i.e., $f\left(t\right)=0$), shown in Figure 2a, can be written as follows:

(8) $$m\ddot{u}+c\dot{u}+ku=0$$

where u, $\dot{u},$ and $\ddot{u}$ are the displacement, velocity, and acceleration of the mass. Dividing Equation (8) by the mass yields:

(9) $$\ddot{u}+2\zeta \omega_{n}\dot{u}+\omega_{n}^{2}u=0$$

where $\omega_{n}=\sqrt{k/m}$ is the undamped natural frequency, and $\zeta =\frac{c}{c_{cr}}=\frac{c}{2\sqrt{km}}$ is the viscous damping ratio [94]. Here, $c_{cr}$ is called the critical damping coefficient. For oscillatory motion (ζ<1) and imposed initial displacement $u_{0}$ and velocity ${\dot{u}}_{0}$, the solution of Equation (9) can be determined as follows [95]:

(10) $$u\left(t\right)=Ae^{-\zeta \omega_{n}t}sin\left(\omega_{d}t+\theta \right)$$

where A and θ are the coefficients to be determined from the initial conditions, and $\omega_{d}=\omega_{n}\sqrt{1-\zeta^{2}}$ is the damped natural frequency. 

#### 2.2.3. Forced Vibrations of a Mass–Spring–Damper System

The equation of motion for a viscously damped SDOF system subjected to a harmonic excitation $f\left(t\right)=F_{0}cos\left(\omega t\right),$ shown in Figure 2a, can be written as follows [96]:

(11) $$m\ddot{u}+c\dot{u}+ku=F_{0}cos\left(\omega t\right)$$

Dividing Equation (11) by the mass yields:

(12) $$\ddot{u}+2\zeta \omega_{n}\dot{u}+\omega_{n}^{2}u=\frac{F_{0}}{m}cos\left(\omega t\right)$$

For oscillatory motion (ζ<1), the solution of Equation (12) can be determined to be the summation of the homogenous solution $u_{h}\left(t\right)$ and particular $u_{p}\left(t\right)$ solution as follows [26]:

(13) $$u\left(t\right)=Ae^{-\zeta \omega_{n}t}sin\left(\omega_{d}t+\theta \right)+Bcos\left(\omega t-\varphi \right)$$

where A and θ are the coefficients to be determined from the initial conditions, and B and φ are the coefficients of the particular solution given by:

(14) $$B=\frac{F_{0}/m}{\sqrt{{\left(\omega_{n}^{2}-\omega^{2}\right)}^{2}+{\left(2\zeta \omega_{n}\omega \right)}^{2}}}$$

(15) $$\varphi ={tan}^{-1}\frac{2\zeta \omega_{n}\omega }{\omega_{n}^{2}-\omega^{2}}$$

The equations above can be further arranged as follows:

(16) $$C=\frac{B}{F_{0}/k}=\frac{1}{\sqrt{{\left(1-r^{2}\right)}^{2}+{\left(2\zeta r\right)}^{2}}}$$

(17) $$\varphi ={tan}^{-1}\frac{2\zeta r}{1-r^{2}}$$

where $r=\omega /\omega_{n}$ is the frequency ratio. Here, φ is the phase lag of the displacement of the mass with respect to the force applied to the mass. It should be remembered that ϕ is the phase lag of the strain with respect to the stress in the material. As presented later, the phase lag of the strain with respect to the stress in the material is $\phi ={tan}^{-1}\left(2\zeta \frac{\omega_{n}}{\omega }\right)={tan}^{-1}\left(2\zeta r\right)$ for a viscously damped system. It should be noted that for the quasistatic loading (i.e., $r=\omega /\omega_{n}\ll 1$), the solution for the forced vibrations of the mass–spring–damper system reduces to that of the system without inertia; hence, we have φ≅ϕ for the quasistatic loading.

### 2.3. Viscously Damped SDOF System Exposed to Step Excitation

The equation of motion for a viscously damped SDOF system subjected to a step excitation $f\left(t\right)=F_{0}$ for t≥0, shown in Figure 2a, can be written as follows:

(18) $$m\ddot{u}+c\dot{u}+ku=F_{0}\;\mathrm{for}\;t\geq 0$$

The response of an underdamped system (ζ<1) exposed to step excitation can be shown as follows [59]:

(19) $$u\left(t\right)=\frac{F_{0}}{k}-\frac{F_{0}}{k\sqrt{1-\zeta^{2}}}e^{-\zeta \omega_{n}t}cos\left(\omega_{d}t-\theta \right)$$

where

(20) $$\theta ={tan}^{-1}\frac{\zeta }{\sqrt{1-\zeta^{2}}}$$

### 2.4. SDOF System with Complex Stiffness Exposed to Harmonic Excitation

#### 2.4.1. Steady-State Response of a Complex Spring System

The equation of motion for a complex spring having real and imaginary components $\tilde{k}=k^{\prime }+jk^{\prime \prime }$ without any inertia (i.e., m=0) exposed to a harmonic excitation $f\left(t\right)=F_{0}sin\left(\omega t\right),$ shown in Figure 2b, can be written as follows:

(21) $$\tilde{k}u=\left(k^{\prime }+jk^{\prime \prime }\right)u=F_{0}sin\left(\omega t\right)$$

where $j=\sqrt{-1}$. The steady-state solution for this system can be shown as follows [38]:

(22) $$u(t)=Bsin\left(\omega t-\phi \right)$$

where

(23) $$B=\frac{F_{0}}{k^{\prime }\sqrt{1+{\left(\frac{k^{\prime \prime }}{k^{\prime }}\right)}^{2}}}=\frac{F_{0}}{k^{\prime }\sqrt{1+{tan}^{2}\phi }}$$

(24) $$\phi ={tan}^{-1}\left(\frac{k^{\prime \prime }}{k^{\prime }}\right)$$

It is worth remembering that the spring with a complex stiffness property is restrained from one end and forced from the other end (see Figure 2b). It is seen that, opposite to the viscously damped system in which the phase angle is a function of the material properties (i.e., c and k) and the frequency (ω), the phase angle is only a function of the material properties for the complex spring system (i.e., $k^{\prime }$ and $k^{\prime \prime }$). However, the material properties can be dependent on the frequency.

#### 2.4.2. Steady-State Response of a Mass–Complex Spring System

The equation of motion for an SDOF system with complex stiffness subjected to harmonic excitation $f\left(t\right)=F_{0}e^{j\omega t}$, shown in Figure 2b, can be written as follows:

(25) $$m\ddot{u}+\left(k^{\prime }+jk^{\prime \prime }\right)u=F_{0}e^{j\omega t}$$

It is common to write $\tilde{k}=k^{\prime }+jk^{\prime \prime }=k+j\eta k=k\left(1+j\eta \right),$ where η is known as the loss factor. Assuming the form of the solution to be as $\tilde{B}e^{j\omega t}$ and substituting this into the equation above, the following expression will be produced:

(26) $$\left[-m\omega^{2}+k\left(1+j\eta \right)\right]\tilde{B}=F_{0}$$

By performing some operations, the equation above can be written as follows:

(27) $$\tilde{B}=\frac{F_{0}/m}{\omega_{n}^{2}-\omega^{2}+j\eta \omega_{n}^{2}}$$

Hence, the amplitude of oscillations and the phase between the displacement of the mass with respect to the force applied to the mass can be written as follows [97]:

(28) $$\left|\tilde{B}\right|=\frac{F_{0}/m}{\sqrt{{\left(\omega_{n}^{2}-\omega^{2}\right)}^{2}+{\left(\eta \omega_{n}^{2}\right)}^{2}}}$$

(29) $$\varphi ={tan}^{-1}\frac{\eta }{1-{\left(\omega /\omega_{n}\right)}^{2}}$$

The equations above can be further arranged as follows:

(30) $$\left|\tilde{C}\right|=\frac{\left|\tilde{B}\right|}{F_{0}/k}=\frac{1}{\sqrt{{\left(1-r^{2}\right)}^{2}+j\eta^{2}}}$$

(31) $$\varphi ={tan}^{-1}\frac{\eta }{1-r^{2}}$$

It is again worth remembering that φ is the phase lag of the displacement of the mass with respect to the force applied to the mass, while ϕ is the phase lag of the strain with respect to the stress in the material. As presented later, the phase lag of the strain with respect to the stress in the material is $\phi ={tan}^{-1}\left(\eta \right)$ for an SDOF system with complex stiffness. As stated before, using the theoretical background presented in Section 2, different damping parameters are derived, and their relationships are presented in Section 3.

## 3. Theoretical Derivations of Different Damping Parameters and Their Relationships

Many techniques are available for the identification of the damping of structures using experimental data. For example, the logarithmic decrement and step-response techniques are time-domain decay-rate methods; the half-power bandwidth, circle-fit, and line-fit methods are frequency-domain modal analysis curve-fitting methods; and the hysteresis loop or power input method is an energy-based technique [95]. Each method gives a different damping parameter (loss factor, viscous damping ratio, etc.). The theoretical derivations of different damping parameters and their relationships as well as damping identification methods are presented in the subsequent sections. The definitions of common damping parameters are listed in Table 2 so that the reader can refer to these parameters as needed.

### 3.1. Hysteresis Loop and Specific Damping Capacity

**Figure 3:**
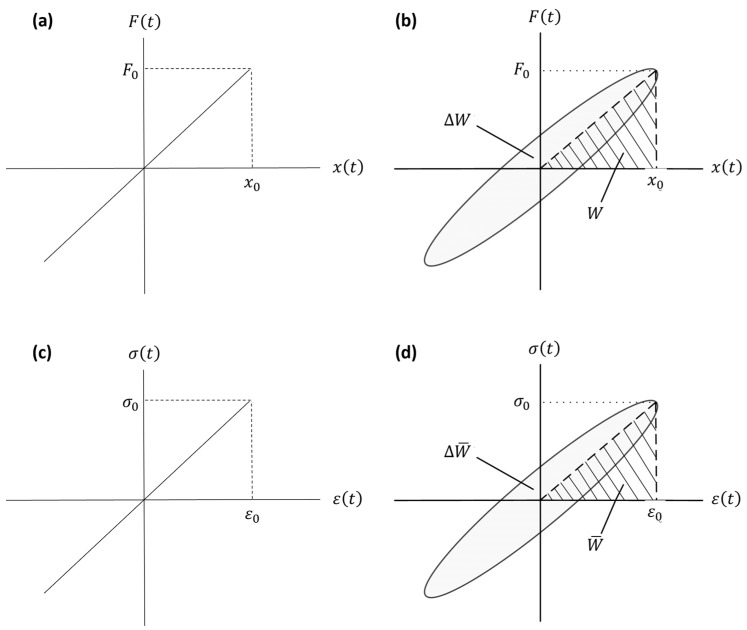
The force–displacement and stress–strain relationships for a purely elastic material (**a**,**c**), and a viscoelastic material (**b**,**d**) under harmonic loading.

The force–displacement and stress–strain relationships for a purely elastic material given in Equation (1) are simply illustrated in Figure 3a,c. As seen, the stress–strain (or force–displacement) curve of a purely elastic material is a straight line. For a viscoelastic material under the cyclic loading at constant frequency ω and for the stress amplitude $\sigma_{0}$ (see Figure 1), the stress can be written as follows:

(32) $$\sigma \left(t\right)=\sigma_{0}e^{j\omega t}$$

The induced strain for a viscoelastic material can be expressed as follows:

(33) $$\varepsilon \left(t\right)=\varepsilon_{0}e^{j\left(\omega t-\phi \right)}$$

where $\varepsilon_{0}$ is the strain amplitude, and, as stated before, ϕ is the phase between the stress and strain. For a viscoelastic material, the input force versus the induced displacement and the input stress $\sigma \left(t\right)$ versus the induced strain $\varepsilon \left(t\right)$ for one cycle of motion are plotted in Figure 3b,d. The elliptical shape shown in Figure 3b,d is known as the hysteresis loop [98,99]. The area captured within the hysteresis loop, ∆W, equals the dissipated energy per cycle of harmonic motion by the material, and W represents the maximum stored energy [93]. Similarly, $∆\bar{W}$ is the energy dissipated per unit volume of the sample during one cycle, and $\bar{W}$ is the maximum stored energy per unit volume. It should be noted that ∆W=0 for a purely elastic material (or spring), while ∆W>0 for a viscoelastic material, and it is proportional to the area bounded by the hysteretic curve. Overall, by harmonically loading a sample in one direction, the hysteresis loop for the sample can be obtained. The specific damping capacity, which is defined as the ratio of the mechanical energy dissipated during one cycle to the maximum potential (strain) energy of the sample, can be calculated using [44]:

(34) $$\psi =\frac{∆W}{W}$$

or

(35) $$\psi =\frac{∆\bar{W}}{\bar{W}}$$

Remembering that the energy dissipated per unit volume of the sample is $∆\bar{W}=\int \sigma d\varepsilon$ and that the maximum energy per unit volume is $\bar{W}=\frac{1}{2}\sigma_{0}\varepsilon_{0}$, the expression above can be written as:

(36) $$\psi =\frac{\oint \sigma d\varepsilon }{\frac{1}{2}\sigma_{0}\varepsilon_{0}}$$

Furthermore, since we can write $\sigma_{0}=E\varepsilon_{0}$, the following expression can be written:

(37) $$\psi =\frac{\oint \sigma d\varepsilon }{\frac{1}{2}E\varepsilon_{0}^{2}}$$

It should be noted that the hysteresis loop method or the power input method is quite effective for determining the frequency-averaged damping of a structure under steady-state vibration.

### 3.2. Hysteresis Loop and Loss Factor

For reasonable levels of damping, the relationship between the structural (or material) damping ratio and associated energy components shown in Figure 3b,d is given by the following equation [93,100]:

(38) $$\eta =\frac{1}{2\pi }\frac{∆W}{W}$$

or

(39) $$\eta =\frac{1}{2\pi }\frac{∆\bar{W}}{\bar{W}}$$

where η is known as the loss factor. Furthermore, using $∆\bar{W}=\int \sigma d\varepsilon$, $\bar{W}=\frac{1}{2}\sigma_{0}\varepsilon_{0}$, and $\sigma_{0}=E\varepsilon_{0}$ in Equation (39) produces the following expression:

(40) $$\eta =\frac{\oint \sigma d\varepsilon }{\pi \sigma_{0}\varepsilon_{0}}$$

or

(41) $$\eta =\frac{\oint \sigma d\varepsilon }{\pi E\varepsilon_{0}^{2}}$$

It is worth reminding that the loss factor is so commonly used to define and quantify the damping of structures in practical applications because it can be easily measured using standard test methods, such as experimental modal analysis [101], is commonly used in modelling damping in the frequency domain [102], can be modelled as a function of frequency [97], is related to the amplitude of the hysteresis loop obtained by cyclic loading of the material [103], and so on.

### 3.3. Specific Damping Capacity and Loss Factor

By substituting Equation (34) into Equation (38), the relationship between the specific damping capacity and loss factor can be easily obtained as follows [104]:

(42) $$\eta =\frac{\psi }{2\pi }$$

It is seen that the loss factor and specific damping capacity are simply related through the constant 1/2π. However, the use of the loss factor provides convenience in modelling damping in structural mechanics [105,106].

### 3.4. Dissipated Energy and Viscous Damping Coefficient

A viscous damper is a velocity-dependent dissipative component that produces damping when the viscous fluid passes through appropriate orifices [107]. The viscous damping force is proportional to the relative velocity between the two ends of the damper ($f=c\dot{u}$, Figure 2a). The viscous damping coefficient c with the unit N s/m is a parameter that represents the energy dissipation due to friction that decelerates motion [108]. For a viscous damper subjected to a harmonic force, the dissipated energy per cycle can be written as follows:

(43) $$∆W=\oint c\dot{x}dx$$

Since $dx=\dot{x}dt$, $x(t)=Bsin\left(\omega t\right)$, and, hence, $\dot{x}(t)=B\omega cos\left(\omega t\right)$, the expression for the dissipated energy per cycle of harmonic motion by the material becomes:

(44) $$∆W=\oint cB^{2}\omega^{2}{cos}^{2}\left(\omega t\right)dt$$

where B is the displacement amplitude. Hence, the integration for the whole cycle produces [36]:

(45) $$∆W=\pi cB^{2}\omega$$

or

(46) $$∆W=2\pi^{2}cB^{2}f$$

Here, f is the frequency in Hz, and ω=2πf is the frequency in rad/s, as stated before. After all, the viscous damping coefficient can be found using the dissipated energy per cycle of harmonic motion as:

(47) $$c=\frac{∆W}{\pi B^{2}\omega }=\frac{∆W}{2\pi^{2}B^{2}f}$$

### 3.5. Complex Modulus and Loss Factor

The Young’s modulus or shear modulus of a viscoelastic material can be represented by a complex (or dynamic) quantity, having both the storage and dissipative energy components. In order to derive the complex modulus, let us write the stress and strain as complex quantities as follows:

(48) $$\sigma \left(t\right)=\sigma_{0}e^{j\omega t}=\sigma_{0}\left[cos\left(\omega t\right)+jsin\left(\omega t\right)\right]$$

(49) $$\varepsilon \left(t\right)=\varepsilon_{0}e^{j\left(\omega t-\phi \right)}=\varepsilon_{0}\left[cos\left(\omega t-\phi \right)+jsin\left(\omega t-\phi \right)\right]$$

where $\sigma_{0}$ and $\varepsilon_{0}$ are the stress and strain amplitudes, respectively. Hence, the complex Young’s modulus $\tilde{E}$ of the material can be written as follows:

(50) $$\tilde{E}=\frac{\sigma_{0}e^{j\omega t}}{\varepsilon_{0}e^{j\left(\omega t-\phi \right)}}=\frac{\sigma_{0}}{\varepsilon_{0}}cos\left(\phi \right)+j\frac{\sigma_{0}}{\varepsilon_{0}}sin\left(\phi \right)=E^{\prime }+jE^{\prime \prime }$$

where $E^{\prime }$ is the storage Young’s modulus, and $E^{\prime \prime }$ is the loss Young’s modulus. The storage Young’s modulus or the real part of the complex Young’s modulus $E^{\prime }=\frac{\sigma_{0}}{\varepsilon_{0}}cos\left(\phi \right)$ is related to the elastic behaviour of the material, and the loss Young’s modulus or the imaginary part of the complex Young’s modulus $E^{\prime \prime }=\frac{\sigma_{0}}{\varepsilon_{0}}sin\left(\phi \right)$ is related to the viscous behaviour of the material (a measure of the energy dissipation ability of the material). When $E^{\prime \prime }=0$, then $E^{\prime }$ takes the place of the ordinary Young’s modulus E. Therefore, it is called the storage Young’s modulus since it measures the material’s ability to store elastic energy. The complex Young’s modulus can be written as follows [109]:

(51) $$\tilde{E}=E^{\prime }\left(1+j\frac{E^{\prime \prime }}{E^{\prime }}\right)$$

The energy dissipated during a load cycle can be written as $∆\bar{W}=\pi E^{\prime \prime }\varepsilon_{0}^{2}$. Similarly, the maximum elastic energy during the cycle can be written as $\bar{W}=\frac{1}{2}E^{\prime }\varepsilon_{0}^{2}$. Substituting these into the equation above yields:

(52) $$\tilde{E}=E^{\prime }\left(1+j\frac{∆W}{2\pi W}\right)$$

Since $\frac{∆W}{2\pi W}$ was defined as the loss factor before, the following expression can be written:

(53) $$\tilde{E}=E^{\prime }\left(1+j\eta \right)$$

From Equations (51) and (53), it is clear that the relationship between the loss factor and the storage and loss Young’s moduli is as follows:

(54) $$\eta =\frac{E^{\prime \prime }}{E^{\prime }}$$

Although the expressions above are written for Young’s modulus, similar expressions can be written for the shear modulus using $\tilde{G}=G^{\prime }+jG^{\prime \prime }$ where $G^{\prime }$ is the storage shear modulus, and $G^{\prime \prime }$ is the loss shear modulus.

### 3.6. Logarithmic Decrement and Viscous Damping Ratio

**Figure 4:**
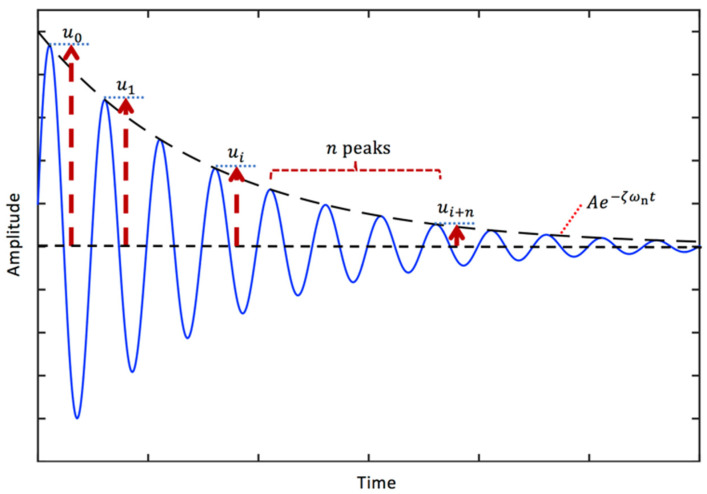
A typical free vibration response of a viscously damped SDOF system (blue solid curve) and the logarithmic decay (black dashed curve).

A typical free vibration response of the viscously damped SDOF system given in Equation (10) is illustrated in Figure 4. It should be noted that the decay envelope for the free vibrations is $Ae^{-\zeta \omega_{n}t}$. The logarithmic decrement is defined as the natural logarithm of the ratio of the amplitudes of any two successive peaks, given by [47]:

(55) $$\delta ={log}_{e}\frac{u_{0}}{u_{1}}={log}_{e}\frac{u_{1}}{u_{2}}={log}_{e}\frac{u_{2}}{u_{3}}=\cdots ={log}_{e}\frac{u_{m-1}}{u_{m}}$$

Using Equations (10) and (55), the following equation that relates the logarithmic decrement and viscous damping ratio can be obtained [110]:

(56) $$\delta =\frac{1}{n}{log}_{e}\frac{u_{i}}{u_{i+n}}=\frac{2\pi \zeta }{\sqrt{1-\zeta^{2}}}$$

where $u_{i}$ is the amplitude of peak i, and $u_{i+n}$ is the amplitude of peak i+n. Overall, the viscous damping ratio using the logarithmic decrement can be determined using the following expression:

(57) $$\zeta =\frac{\delta }{\sqrt{4\pi^{2}+\delta^{2}}}$$

Since $\sqrt{1-\zeta^{2}}\approx 1$ for small damping, it is common to define the relationship between the viscous damping ratio and logarithmic decrement as follows:

(58) $$\zeta_{appr}=\frac{\delta }{2\pi }\;\mathrm{for}\;\zeta \ll 1$$

The logarithmic decrement method is a time-domain identification approach that does not require input measurement; it requires only response measurements. It should be noted that the logarithmic decrement method is effective for damping identification when a single mode of vibration can be isolated from the others.

**Figure 5:**
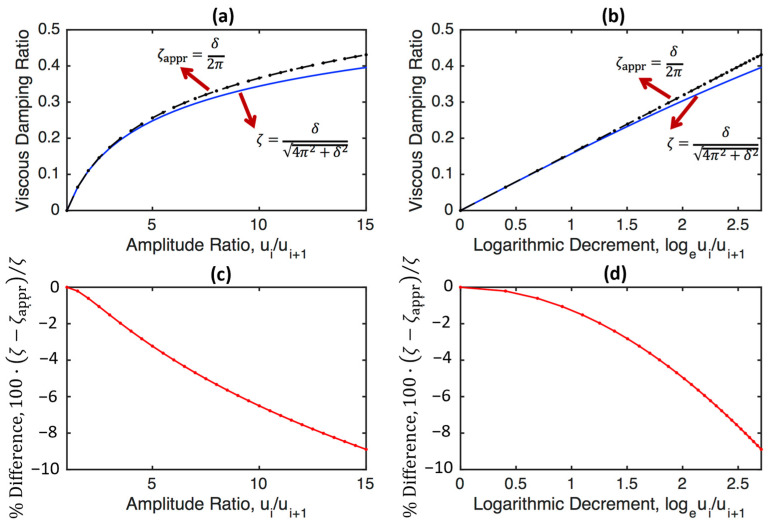
The real viscous damping ratio, calculated using Equation (57), and the approximate viscous damping ratio, calculated using Equation (58), as a function of the amplitude ratio (**a**), the logarithmic decrement (**b**), the percentage difference as a function of the amplitude ratio (**c**), and the logarithmic decrement (**d**).

Figure 5 presents the real viscous damping ratio, calculated using Equation (57), and the approximate viscous damping ratio, calculated using Equation (58), as a function of the amplitude ratio (Figure 5a), the logarithmic decrement (Figure 5b), the percentage difference as a function of the amplitude ratio (Figure 5c), and the logarithmic decrement (Figure 5d). It is seen that the difference is greater than 2% when the amplitude ratio is greater than or equal to 3.5 or when the logarithmic decrement is greater than or equal to 1.25. As the exact formula (i.e., Equation (57)) is still quite simple, the use of Equation (57) is highly recommended when calculating the viscous damping ratio from the logarithmic decrement in practical applications.

### 3.7. Half-Power Bandwidth and Viscous Damping Ratio

**Figure 6:**
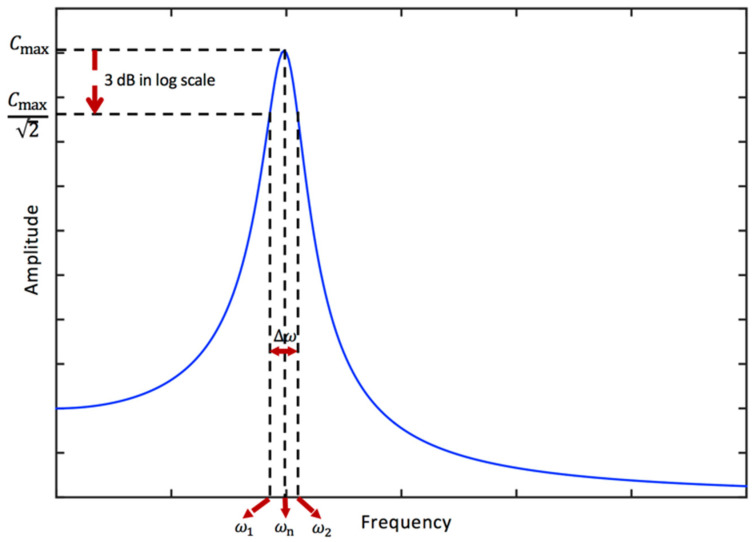
The illustration for the procedure for the use of a half-power bandwidth for the identification of damping.

For a viscously damped SDOF system subjected to a harmonic excitation, the contribution of the homogenous (or transient) solution (i.e., the response due to the initial conditions) is diminished for large values of t [26]; hence, we have only the particular solution (i.e., the response due to the applied force) at the steady state (see Equation (13)). As the power is proportional to the square of the amplitude of oscillations, the half-power response level corresponds to $B_{max}/\sqrt{2}$, where $B_{max}$ is the maximum value of the amplitude B given in Equation (14). The half-power frequencies are the two points on either side of the natural frequency such that the dynamic amplification is equal to $1/\sqrt{2}$. It should be noted that this corresponds to a 3 dB amplitude decrease in the logarithmic scale. The procedure for the use of a half-power bandwidth for the identification of damping is illustrated in Figure 6. Using Equations (14) and (16), the operation $\frac{d}{dr}\left(\frac{B}{F_{0}/k}\right)=0$ produces the peak value $C_{max}=\frac{1}{2\zeta \sqrt{1-\zeta^{2}}}$ at $r_{max}=\sqrt{1-{2\zeta }^{2}}$ [26]. Hence, using $C\left(r\right)=C_{max}/\sqrt{2}$ in Equation (16) yields the frequency ratios at the half-power points as $r_{1,2}=\sqrt{1-{2\zeta }^{2}\pm 2\zeta \sqrt{1+\zeta^{2}}}$. Hence, the lower and upper half-power frequencies are obtained as $\omega_{1}=\omega_{n}\sqrt{1-{2\zeta }^{2}-2\zeta \sqrt{1+\zeta^{2}}}$ and $\omega_{2}=\omega_{n}\sqrt{1-{2\zeta }^{2}+2\zeta \sqrt{1+\zeta^{2}}}$, respectively. Overall, the relationship between the viscous damping ratio and half-power bandwidth becomes as follows [38,111]:

(59) $$\frac{∆\omega }{\omega_{n}}=\frac{\omega_{2}-\omega_{1}}{\omega_{n}}=\sqrt{1-{2\zeta }^{2}+2\zeta \sqrt{1+\zeta^{2}}}-\sqrt{1-{2\zeta }^{2}-2\zeta \sqrt{1+\zeta^{2}}}$$

where $∆\omega =\omega_{2}-\omega_{1}$ is the half-power bandwidth. Once the half-power frequencies and natural frequency are determined using the measured data, the viscous damping ratio can be found using Equation (59). It is clear that the wider bandwidth means more damping. The expression in Equation (59) is quite complicated. Therefore, the following approximate expression can be used to define the relationship between the half-power bandwidth and viscous damping ratio:

(60) $$\frac{∆\omega }{\omega_{n}}=\frac{\omega_{2}-\omega_{1}}{\omega_{n}}=\sqrt{1+2\zeta }-\sqrt{1-2\zeta }\;\mathrm{for}\;\zeta <0.1$$

For small damping (i.e., ζ≪1), the equation can be further simplified as follows [112]:

(61) $$\frac{∆\omega }{\omega_{n}}=\frac{\omega_{2}-\omega_{1}}{\omega_{n}}=2\zeta_{appr}\;\mathrm{for}\;\zeta \ll 1$$

**Figure 7:**
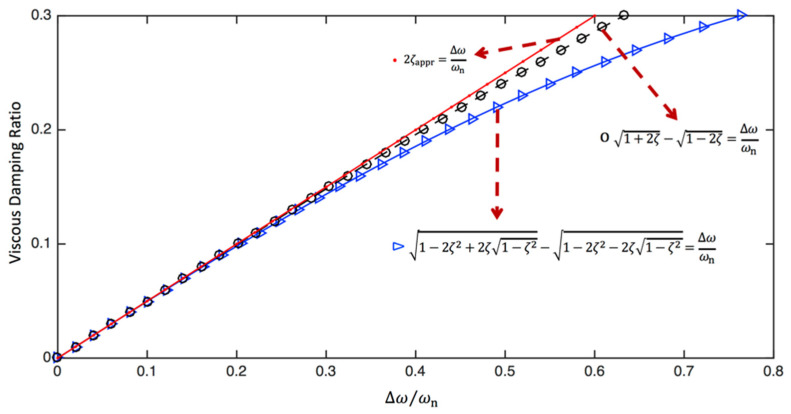
The relationship between the half-power bandwidth and viscous damping ratio calculated using Equations (59)–(61).

The relationship between the half-power bandwidth and the viscous damping ratio using Equations (59)–(61) is visualised in Figure 7. It is seen that for small damping (i.e., $\frac{∆\omega }{\omega_{n}}<0.2$ or ζ<0.1), Equations (59)–(61) produce almost the same results, whereas there is a considerable difference between Equation (59) and Equations (60) and (61) when ζ>0.2. On the other hand, the approximation given in Equation (60) is always better than the simplest expression given in Equation (61).

### 3.8. Half-Power Bandwidth and Loss Factor

As will be shown later, the loss factor can be written as $\eta =tan\left(\phi \right)$. Hence, the relationship between the loss factor and half-power bandwidth can be shown as [38]:

(62) $$\frac{∆\omega }{\omega_{n}}=\frac{\omega_{2}-\omega_{1}}{\omega_{n}}=\sqrt{1+\eta }-\sqrt{1-\eta }$$

For small and medium damping, the equation can be further simplified as:

(63) $$\frac{∆\omega }{\omega_{n}}=\frac{\omega_{2}-\omega_{1}}{\omega_{n}}=\eta \;\mathrm{for}\;\eta <0.3$$

**Figure 8:**
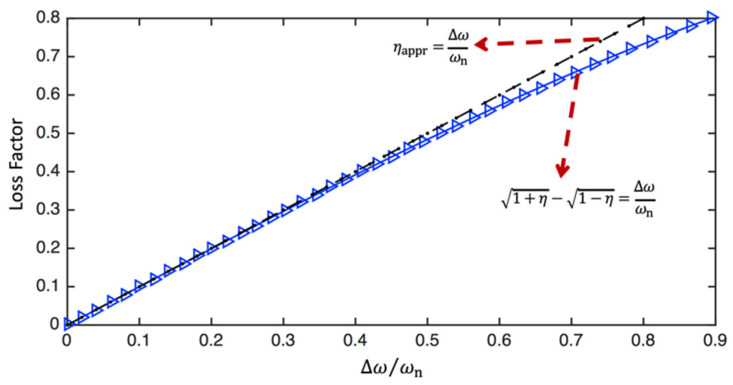
The relationship between the half-power bandwidth and loss factor calculated using Equations (62) and (63).

The relationship between the half-power bandwidth and loss factor calculated using Equations (62) and (63) is visualised in Figure 8. It is seen that for small and medium damping (i.e., $\frac{∆\omega }{\omega_{n}}<0.3$ or η<0.3), Equations (62) and (63) produce almost the same results, whereas there is a considerable difference between Equations (62) and (63) when η>0.6. 

In practical applications, the most commonly used methods for the identification of the loss factor require vibration spectrums or frequency response functions, which are obtained via the Fourier transformation of the time-domain data [113]. Although, we presented the half-power bandwidth concept above, more sophisticated methods such as the circle-fit and line-fit methods are commonly used to identify the modal loss factors of a structure using measured frequency response functions [95,97,114,115]. 

### 3.9. Loss Factor and Viscous Damping Ratio

As seen in Equation (54), the loss factor is defined as $\eta =\frac{E^{\prime \prime }}{E^{\prime }}$ for the complex Young’s modulus $E^{\prime }+jE^{\prime \prime }$. It should be remembered that, similarly, the same concept is used for the complex stiffness given by $\tilde{k}=k^{\prime }+jk^{\prime \prime }$, where $k^{\prime }$ and $k^{\prime \prime }$ are the real and imaginary parts of the complex stiffness, respectively. Let us try to obtain the equivalent complex stiffness for a viscously damped SDOF system. The equation of motion for a viscously damped SDOF system subjected to a harmonic excitation can be written as:

(64) $$m\ddot{u}+c\dot{u}+ku=F_{0}e^{j\omega t}$$

Assuming the form of the solution to be $\tilde{B}e^{j\omega t}$ and substituting this into Equation (64) produces the following expression:

(65) $$\left[-m\omega^{2}+\left(k+jc\omega \right)\right]\tilde{B}=F_{0}$$

Equation (65) can be further arranged as follows:

(66) $$\left[-m\omega^{2}+k\left(1+j\frac{c\omega }{k}\right)\right]\tilde{B}=F_{0}$$

or

(67) $$\left(-m\omega^{2}+\tilde{k}\right)\tilde{B}=F_{0}$$

where $\tilde{k}$ is the complex stiffness defined as:

(68) $$\tilde{k}=k^{\prime }+jk^{\prime \prime }=k\left(1+j\frac{c\omega }{k}\right)$$

Hence, the loss factor for a viscously damped system can obtained as follows [38]:

(69) $$\eta =\frac{k^{\prime \prime }}{k^{\prime }}=\frac{c\omega }{k}$$

Using the definitions of $\omega_{n}=\sqrt{k/m}$, and $\zeta =\frac{c}{c_{cr}}=\frac{c}{2\sqrt{km}}$ at $\omega =\omega_{d}$, the relationship between the loss factor and viscous damping ratio is obtained as:

(70) $$\eta =2\zeta \sqrt{1-\zeta^{2}}$$

Since $2\zeta \sqrt{1-\zeta^{2}}\approx 2\zeta$ for small damping, the equation above can be written as:

(71) $$\eta_{appr}=2\zeta \;\mathrm{for}\;\zeta \ll 1$$

**Figure 9:**
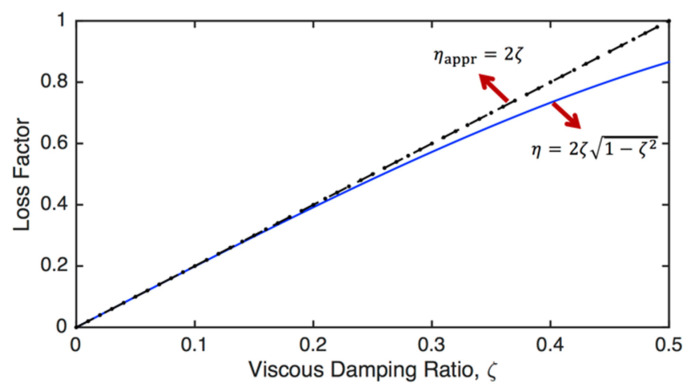
The relationship between the viscous damping ratio and loss factor, calculated using Equations (70) and (71).

The relationship between the viscous damping ratio and loss factor calculated using Equations (70) and (71) is visualised in Figure 9. It is seen that for small damping (i.e., ζ<0.15), both approaches produce almost the same results, whereas there is a considerable difference between these two approaches when ζ>0.3. 

### 3.10. Phase Lag and Loss Factor

As seen in Equation (50), the storage and loss moduli are given by $E^{\prime }=\frac{\sigma_{0}}{\varepsilon_{0}}cos\left(\phi \right)$ and $E^{\prime \prime }=\frac{\sigma_{0}}{\varepsilon_{0}}sin\left(\phi \right)$, respectively. Using these in Equation (54), the relationship between the phase lag and loss factor is obtained as [116]:

(72) $$\eta =\frac{E^{\prime \prime }}{E^{\prime }}=tan\left(\phi \right)$$

Hence, the phase lag in terms of the loss factor can be written as follows:

(73) $$\phi ={tan}^{-1}\left(\eta \right)$$

### 3.11. Phase Lag and Viscous Damping Ratio

Using $\omega_{n}=\sqrt{k/m}$ and $\zeta =\frac{c}{c_{cr}}=\frac{c}{2\sqrt{km}}$ in Equation (7), the relationship between the phase lag and viscous damping ratio can be shown as:

(74) $$2\zeta \frac{\omega_{n}}{\omega }=tan\left(\phi \right)$$

Hence, the phase lag in terms of viscous damping ratio can be written as:

(75) $$\phi ={tan}^{-1}\left(2\zeta \frac{\omega_{n}}{\omega }\right)$$

At $\omega =\omega_{n}$, the relation between the loss angle and viscous damping ratio becomes:

(76) $$\phi ={tan}^{-1}\left(2\zeta \right)\;\mathrm{at}\;\omega =\omega_{n}$$

### 3.12. Viscosity and Loss Modulus

In the oscillatory shear experiment, the rotation provided to the sample is a simple harmonic motion. Hence, the shear strain can be written as [117]:

(77) $$\gamma \left(t\right)=\gamma_{0}sin\left(\omega t\right)$$

where $\gamma_{0}$ and ω are the input strain amplitude and frequency, respectively. Based on Equation (77), the shear strain rate will be:

(78) $$\dot{\gamma }\left(t\right)=\frac{d\gamma }{dt}=\gamma_{0}\omega cos\left(\omega t\right)$$

For a linear viscoelastic material, the stress response to the applied shear is determined not only by the current rate of strain but also by the historical rate of strain. Hence, the stress for a general linear viscoelastic material at time t can be written as [118,119]:

(79) $$\tau \left(t\right)=\int_{-\infty }^{t}G\left(t-t^{\prime }\right)\dot{\gamma }\left(t^{\prime }\right)dt^{\prime }=\int_{-\infty }^{t}G\left(t-t\prime \right)\gamma_{0}\omega cos\left(\omega t\prime \right)dt^{\prime }$$

where the function G(t) is the relaxation modulus of the fluid [120] and shows the importance of the past strain rate on the current stress in the system. It is worth noticing that a linear elastic solid has a constant relaxation modulus of $G\left(t\right)=G_{0}$, and a purely viscous fluid has a relaxation modulus of $G\left(t\right)=\mu \delta \left(t\right),$ where μ is the viscosity, and $\delta \left(t\right)$ is the Dirac delta function [118]. Overall, using reference [121], the relationship between the loss modulus and viscosity is obtained, as explained below. First, by changing the variables using ${s=t-t}^{\prime }$, we can transform the integral in Equation (79) to the following expression:

(80) $$\tau \left(t\right)=\gamma_{0}\omega \int_{0}^{\infty }G\left(s\right)cos\left[\omega \left(t-s\right)\right]ds$$

In addition, by writing $cos\left[\omega \left(t-s\right)\right]=Re\left[e^{j\omega \left(t-s\right)}\right]$, we can obtain the following equation:

(81) $$\tau \left(t\right)=\gamma_{0}\omega \int_{0}^{\infty }G\left(s\right)Re\left[e^{j\omega \left(t-s\right)}\right]ds=\gamma_{0}\omega Re\left[e^{j\omega t}\int_{0}^{\infty }G\left(s\right)e^{-j\omega s}ds\right]$$

It is clear that the integral above is a one-sided Fourier transformation, and since it has no dependence on t, it is a complex number. By convention, we can define the complex shear modulus $\tilde{G}$ as follows:

(82) $$\tilde{G}=j\omega \int_{0}^{\infty }G\left(s\right)e^{-j\omega s}ds=G^{\prime }+jG\prime \prime$$

where $G^{\prime }$ is the storage shear modulus, and $G^{\prime \prime }$ is the loss shear modulus, as stated before. Overall, we have the following expression:

(83) $$\tau \left(t\right)=\gamma_{0}Re\left[e^{j\omega t}\left(-j\tilde{G}\right)\right]=\gamma_{0}Re\left[cos\left(\omega t\right)+jsin\left(\omega t\right)\left(G^{\prime \prime }-jG^{\prime }\right)\right]$$

By further rearranging the expression above and substituting Equations (77) and (78) into Equation (83), we can obtain the following equation [118]:

(84) $$\tau \left(t\right)=\gamma_{0}\left[G^{\prime }sin\left(\omega t\right)+G\prime \prime cos\left(\omega t\right)\right]=G^{\prime }\gamma \left(t\right)+\frac{G^{\prime \prime }}{\omega }\dot{\gamma }\left(t\right)$$

It should be noted that the response of a purely viscous fluid is $\tau \left(t\right)=\mu \dot{\gamma }\left(t\right)=\mu \gamma_{0}\omega cos\left(\omega t\right)$, and the response of a purely elastic solid is $\tau \left(t\right)$ $=G\gamma \left(t\right)=G\gamma_{0}sin\left(\omega t\right)$. As seen in Equation (84), the role of the viscosity is found using the term $\frac{G^{\prime \prime }}{\omega }$. Therefore, it is common to write the shear viscosity in terms of shear loss modulus as follows:

(85) $$\mu =\frac{G^{\prime \prime }}{\omega }$$

### 3.13. Viscosity and Loss Factor

As the loss factor is defined as $\eta =\frac{G^{\prime \prime }}{G^{\prime }}$, using Equation (85), the viscosity in terms of the loss factor can be written as follows [122]:

(86) $$\mu =\frac{\eta G^{\prime }}{\omega }$$

### 3.14. Inverse Quality Factor and Viscous Damping Ratio

The inverse quality factor for a mechanical system is defined as the inverse of the so-called quality factor (Q), and using Equation (59), it can be written as follows [38]:

(87) $$Q_{inv}=\frac{1}{Q}=\frac{∆\omega }{\omega_{n}}=\sqrt{1-{2\zeta }^{2}+2\zeta \sqrt{1+\zeta^{2}}}-\sqrt{1-{2\zeta }^{2}-2\zeta \sqrt{1+\zeta^{2}}}$$

For small damping, Equation (87) can be written as follows:

(88) $$Q_{inv,appr}=\frac{1}{Q}=\frac{∆\omega }{\omega_{n}}=2\zeta \;\mathrm{for}\;\zeta \ll 1$$

### 3.15. Inverse Quality Factor and Loss Factor

The inverse quality factor in terms of the loss factor is given by [38]:

(89) $$Q_{inv}=\frac{1}{Q}=\frac{∆\omega }{\omega_{n}}=\sqrt{1+\eta }-\sqrt{1-\eta }$$

For small and medium damping, Equation (89) can be written as follows:

(90) $$Q_{inv,appr}=\frac{1}{Q}=\frac{∆\omega }{\omega_{n}}=\eta \;\mathrm{for}\;\eta <0.3$$

### 3.16. Structural Reverberation Time and Loss Factor

**Figure 10:**
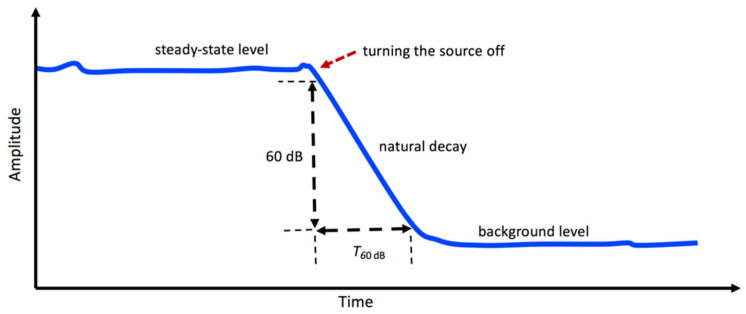
Illustration of the measurement procedure for the 60 dB decay time (*T*_60 dB_).

The loss factor of a plate-like structure can be identified using the method based on the energy attenuation [123]. For this purpose, the structure is suspended by a set of soft springs, and then it is excited by a shaker. When the steady vibrations are set, the excitation is abruptly interrupted, and the decay time of the vibrations is measured (see Figure 10). Hence, the loss factor of the plate is estimated using the following expression [124,125,126]:

(91) $$\eta =\frac{2.2}{f\cdot T_{60\;dB}}=\frac{6{log}_{e}10}{\omega \cdot T_{60\;dB}}$$

where f is the frequency in Hz, ω=2πf is the frequency in rad/s, as stated before, and $T_{60\;dB}$ is the 60 dB decay time (see Figure 10) or structural reverberation time in s.

### 3.17. Step Response and Viscous Damping Ratio

**Figure 11:**
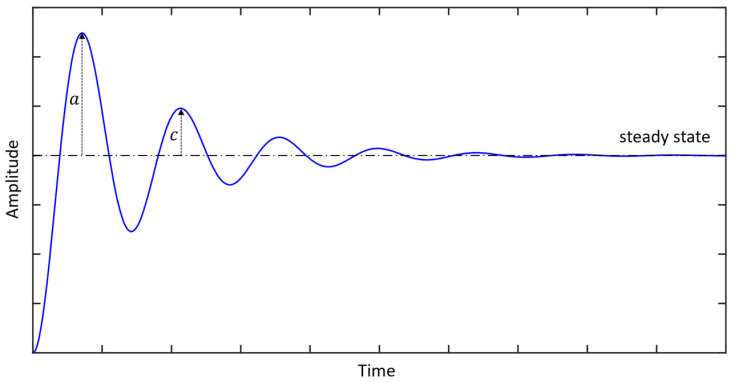
The typical step response of a viscously damped SDOF system.

A typical step response of the viscously damped SDOF system given in Equation (19) is illustrated in Figure 11. Various parameters of the step response, such as the so-called peak time, rise time, overshoot, decay ratio, and settling time can be related to the viscous damping ratio [127]. For example, the relationship between the viscous damping ratio ζ and the decay ratio γ=c/a can be shown as:

(92) $$\zeta =\frac{-{log}_{e}\gamma }{\sqrt{4\pi^{2}+{\left({log}_{e}\gamma \right)}^{2}}}$$

where a and c are the amplitudes of the first and second peaks, respectively.

### 3.18. Rayleigh Damping and Viscous Damping

A common method of modelling damping in practical applications is the so-called Rayleigh damping [128]. It is usually known as proportional damping or classical damping [129]. Overall, the Rayleigh damping model approximates the viscous damping available in the system. In this model, two damping coefficients (i.e., α and β) are specified. These coefficients can be calculated from the modal viscous damping ratio $\zeta_{n}$ at a particular frequency $\omega_{n}$ using the following simple expression [130]:

(93) $$\zeta_{n}=\frac{\alpha }{2\omega_{n}}+\frac{\beta \omega_{n}}{2}$$

If the viscous damping ratios for the ith and jth modes are $\zeta_{i}$ and $\zeta_{j}$, then the Rayleigh coefficients α and β are determined from the solution of the following two algebraic equations [131]:

(94) $$\frac{1}{2}\left[\begin{array}{cc} 1/\omega_{i} & \omega_{i}\\ 1/\omega_{j} & \omega_{j} \end{array}\right]\left[\begin{array}{c} \alpha \\ \beta \end{array}\right]=\left[\begin{array}{c} \zeta_{i}\\ \zeta_{j} \end{array}\right]$$

If both modes have the same viscous damping ratio (i.e., $\zeta_{i}=\zeta_{j}=\zeta$), then the values of α and β can be determined as follows:

(95) $$\alpha =\zeta \frac{2\omega_{i}\omega_{j}}{\omega_{i}+\omega_{j}}\;\mathrm{and}\;\beta =\zeta \frac{2}{\omega_{i}+\omega_{j}}$$

It is worth noting that the Rayleigh damping model is implemented in many finite element software packages, including ABAQUS [132], ANSYS [133], and COMSOL [134].

## 4. Summary of the Relationships between Common Damping Parameters

In practical applications, often one of the damping parameters (e.g., loss factor) is measured, and for comparison purposes, it is necessary to convert the measured damping parameter into some other damping parameter (e.g., viscosity). The measured parameter can be converted into the desired parameter using the expressions presented in Section 3. Using the derived expressions in Section 3, an important equation relating the loss factor (η) to the ratio of the dissipated energy per cycle (∆W) and maximum stored energy (W), specific damping capacity (ψ), loss angle (ϕ), ratio of the loss modulus ($E^{\prime \prime }$) and storage modulus ($E^{\prime }$), and viscous damping ratio (ζ) can be written as follows:

(96) $$\eta =\frac{1}{2\pi }\frac{∆W}{W}=\frac{\psi }{2\pi }=tan\left(\phi \right)=\frac{E^{\prime \prime }}{E^{\prime }}=2\zeta \sqrt{1-\zeta^{2}}$$

Again, using the derived expressions in Section 3, for small damping, another important equation relating the viscous damping ratio (ζ) to the ratio of the dissipated energy per cycle (∆W) and maximum stored energy (W), specific damping capacity (ψ), loss angle (ϕ), ratio of the loss modulus ($E^{\prime \prime }$) and storage modulus ($E^{\prime }$), loss factor (η), logarithmic decrement (δ), ratio of the half-power bandwidth (∆ω) and natural frequency ($\omega_{n}$), quality factor (Q), and inverse quality factor ($Q_{inv}$) at $\omega =\omega_{n}$ can be written as follows:

(97) $$\zeta =\frac{1}{4\pi }\frac{∆W}{W}=\frac{\psi }{4\pi }=\frac{tan\left(\phi \right)}{2}=\frac{E^{\prime \prime }}{2E^{\prime }}=\frac{\eta }{2}=\frac{\delta }{2\pi }=\frac{∆\omega }{2\omega_{n}}=\frac{1}{2Q}=\frac{Q_{inv}}{2}\;\mathrm{for}\;\zeta \ll 1\;(\mathrm{at}\;\omega =\omega_{n})$$

Overall, the important damping parameters measured in practical applications and their relations to other important damping parameters are summarised in Table 3.

## 5. Some Damping Identification Applications of Biomaterials

The dynamic indentation test is widely used to identify the viscoelastic properties of biomaterials. For example, the dynamic indentation method was used to determine the storage and loss moduli of some agar samples [30]. The average storage modulus ($E^{\prime }$) and loss modulus ($E^{\prime \prime }$) for a 5% agar sample obtained using the frequency sweep load function with a 1500 μN static load and 2 μN dynamic amplitude were found to be between 2 and 2.3 MPa and 0.013 and 0.02 MPa, respectively, in the frequency range of 100–200 Hz [30]. Using Equation (54), i.e., $\eta =\frac{E^{\prime \prime }}{E^{\prime }}$, the loss factor of the 5% agar sample can be calculated to be around 0.07 and 0.09 at 100 and 200 Hz, respectively.

It is quite common to measure the storage and loss shear moduli of soft materials using an oscillatory rheometer and then calculate the loss factor or viscosity from the measured storage and loss shear moduli. For instance, the storage shear modulus ($G^{\prime }$) and loss shear modulus ($G^{\prime \prime }$) of a hydrogel were measured using an oscillatory rheometer test [135]. Using the relationship between the loss factor and the storage and loss shear moduli given before (i.e., $\eta =G^{\prime \prime }/G^{\prime }$), the average loss factor of the hydrogel for the given frequency range (i.e., 1–10 Hz) can be calculated to be η=0.007. Similarly, using the relationship between the viscosity and loss shear modulus (i.e., $\mu =G^{\prime \prime }/\omega$) and the given frequency, the viscosity of the hydrogel at f=10 Hz can be calculated to be μ=4 Pa·s.

The logarithmic decrement method is effective for determining the damping of a structure when a single mode of vibration can be isolated from the others. Furthermore, this time-domain method does not require input measurement; it requires only response measurements. For example, the vibration damping characteristics of some spider silk threads were determined through the nanoindentation and the time decay waveform obtained from a laser vibrometer [136]. Using the measured time decay waveform and Equation (56), the logarithmic decrement of the so-called spiral thread was calculated. Then, the viscous damping ratio of the spiral thread was calculated using Equation (57). It should be noted that although the measured time decay waveform given in [136] is not purely harmonic, it is still dominated by a frequency component, and the logarithmic decrement can be used to identify the damping of the structure. Overall, the viscous damping ratio for the spiral thread was found to be ζ=0.12 [136]. Using Equation (71), i.e., η=2ζ, the loss factor of the spiral thread can be calculated to be η=0.24. 

The resonant vibration test or experimental modal analysis is quite commonly used to identify the damping of a structure. The viscous damping ratios of some hydrogel beam-shaped samples were identified using resonant vibration tests for the first bending mode [33]. For this purpose, the frequency response functions using an accelerometer and a laser Doppler vibrometer were measured. The modal viscous damping ratio was determined by fitting the Euler–Bernoulli beam model to the experimental data. Using Equation (72), i.e., $\eta =\frac{E^{\prime \prime }}{E^{\prime }}=tan\left(\phi \right)$, the loss factor of the hydrogel sample was determined, and using the simplified relation between the loss factor and viscous damping ratio (i.e., η=2ζ), the viscous damping ratio of the hydrogel sample was calculated. For example, the viscous damping ratio for the hydrogel 0.8% Bis sample was found to be ζ=0.019 [33]. 

As mentioned before, although the half-power bandwidth concept for the identification of the loss factor was presented in Section 3, more sophisticated methods such as the circle-fit and line-fit methods are commonly used to identify the modal loss factors of a structure using the measured frequency response functions [97]. For instance, the circle-fit model is based on fitting a circle to the measured frequency response function data around the vicinity of a natural frequency. Although the viscous damping ratio can be identified using Equations (59)–(61) based on the half-power bandwidth method, the modal loss factor for the r^th^ mode ($\eta_{r}$) of a structure is determined using $\eta_{r}=\frac{\omega_{2,r}^{2}-\omega_{1,r}^{2}}{\omega_{n,r}^{2}\left[tan\left(\theta_{2,r}/2\right)+tan\left(\theta_{1,r}/2\right)\right]}$ in the circle-fit method, where $\omega_{n,r}$ is the natural frequency of the r^th^ mode, and $\omega_{r,1}$ and $\omega_{r,2}$ correspond to the angles $\theta_{r,1}$ and $\theta_{r,2}$ around $\omega_{n,r}$ when the frequency response function is plotted using the Nyquist diagram. For example, the loss factor of a biofibre-based plate for the first mode using the circle-fit method was determined to be η=0.027 [114]. Using the simplified relation between the loss factor and viscous damping ratio (i.e., η=2ζ), the viscous damping ratio of the biofibre-based plate can be determined to be ζ=0.0135. 

In the recent years, a bubble or sphere placed inside the soft medium [73,74,75] or located at the soft medium interface [34,60,61] and exposed to an external excitation, such as acoustic radiation force or magnetic force, has been widely used to identify the viscoelastic properties of soft materials. For instance, by using the deformation curve for a microbubble administered into a wall-less hydrogel channel that is exposed to an acoustic pulse obtained via high-speed microscopy and the curve fitted to the measured deformation curve exploiting a mathematical model, the viscosity of a gel was estimated [34]. Overall, the maximum displacement of the bubble was determined to be around 2.2 μm, and the viscosity of the hydrogel was estimated to be 0.12 Pa·s [34]. Using a novel approach based on the dynamic response of a spherical object placed at the sample interface, the shear modulus and viscosity of a gelatine sample with a density of 1105 kg/m^3^ were determined to be 3000 Pa and 1.5 Pa⋅s, respectively [60]. 

An ultrasound elastography for the characterisation of the viscoelastic properties of soft tissue was developed and validated [64]. Reverberant shear wave ultrasound elastography was used to scan plantar soft tissue and gelatine phantoms at 400–600 Hz. The shear wave speed was determined using the ultrasound particle velocity data. The viscoelastic parameters were extracted by fitting Young’s modulus as a function of the frequency derived using different rheological models to the shear wave dispersion data. For example, Young’s modulus and the viscosity of plantar soft tissue were determined to be 13,628 Pa and 3.3 Pa⋅s, respectively, using the Kelvin–Voight model [64]. It should be noted that there have been many attempts to exploit damping (or viscosity) in quantitative ultrasound [62,64,137,138,139,140]. For example, the reconstructions of viscosity maps in different tissues (e.g., ex vivo normal porcine liver, fatty duck liver, and fatty goose liver) with inclusions were presented in [64]. In addition, modifications have been made to existing magnetic resonance elastography by using a damping parameter (e.g., loss angle) to improve its accuracy [45,67,80,141,142].

## 6. Conclusions

The literature review shows that the dynamic indentation method, rheometry and viscometry, atomic force microscopy, hysteresis loop or power input method, resonant vibration tests or experimental modal analysis, and logarithmic decrement are commonly used to identify the damping of materials, including soft materials. In addition, a bubble or sphere placed inside the soft medium or located at the soft medium interface while being exposed to an external excitation, such as acoustic radiation force or magnetic force, is nowadays used to identify the viscoelastic properties of soft materials. The use of ultrasound elastography and magnetic resonance elastography for determining the mechanical properties of tissue are quite common in preclinical and clinical applications. The viscous damping ratio, loss factor, complex modulus (or storage and loss moduli), and viscosity are quite commonly used to describe and quantify damping in practical applications. In addition, the specific damping capacity, loss angle, half-power bandwidth, logarithmic decrement, and inverse quality factor are used to describe and quantify damping in many applications. In practice, usually one of the damping parameters (e.g., loss factor) is measured, and for comparison purposes, the measured damping parameter needs to be converted into some other damping parameters (e.g., viscosity). 

There are a limited number of review studies in the literature that present the theoretical derivations of different damping parameters and the relationships between a large number of damping parameters. Therefore, the theoretical derivations of many parameters for the description and quantification of damping as well as their relationships are covered in this comprehensive review. Both accurate formulas (i.e., for systems with any amount of damping) and approximate formulas (i.e., for systems with low damping) are presented and compared. This is the first comprehensive review paper of its kind that presents the theoretical derivations of a large number of damping parameters, and the relationships between many damping parameters with the quantitative evaluation of accurate and approximate formulas. The damping parameters investigated in this paper include the specific damping capacity, loss factor, viscous damping coefficient, viscous damping ratio, loss angle or phase lag, logarithmic decrement, half-power bandwidth, complex modulus (or loss and storage moduli), inverse quality factor, viscosity, decay ratio in the step response, and structural reverberation time. It is believed that the material presented in this paper will be a primary resource for damping or viscoelasticity research and teaching in the future.

## Figures and Tables

**Table 1:** The damping parameters of various materials identified using different methods in the literature.

Material	Method	Damping Parameter	Reference, Year
Agar gels	Dynamic indentation	Complex modulus	Nayar et al. [30], 2012
Human skin	Dynamic indentation	Viscous damping coefficient	Boyer et al. [49], 2009
Cells	Atomic force microscope	Complex modulus	Cartagena-Rivera et al. [50], 2015
Cells	Rheometer	Complex modulus	Dakhil et al. [31], 2016
Cellulose nanocrystal dispersions	Capillary viscometer	Viscosity	Peng et al. [32], 2018
Polyacrylamide gels	Resonant vibration test	Viscous damping ratio	Wang et al. [33], 2020
Silicon rubber samples	Hysteresis loop	Viscous damping coefficient	Esmaeel et al. [36], 2021
Microsperma	Vibration test	Logarithmic decrement	Miao et al. [47], 2021
Gelatine phantom	Acoustic particle palpation	Viscosity	Bezer et al. [34], 2021
Human aorta	Hysteresis loop	Loss factor	Shahmansouri et al. [51], 2016
Bovine nucleus pulposus	Hysteresis loop	Specific damping capacity	Vogel and Pioletti [52], 2012
Human liver	Ultrasound elastography	Viscosity	Chen at al. [69], 2013
Human liver	Magnetic resonance elastography	Loss angle	Wang et al. [67], 2024

**Table 2:** The definitions of common damping parameters.

Parameter	Symbol	Definition/Explanation	
Specific damping capacity	ψ	$\psi =\frac{∆W}{W}$	∆W: area captured within the hysteresis loop W: maximum stored energy
Loss factor	η	$\eta =\frac{1}{2\pi }\frac{∆W}{W}$	
Complex Young’s modulus (unit: Pa)	$\tilde{E}$	$\tilde{E}=E^{\prime }+jE^{\prime \prime }$	$E^{\prime }$: storage Young’s modulus $E^{\prime \prime }$: loss Young’s modulus
Complex shear modulus (unit: Pa)	$\tilde{G}$	$\tilde{G}=G^{\prime }+jG^{\prime \prime }$	$G^{\prime }$: storage shear modulus $G^{\prime \prime }$: loss shear modulus
Logarithmic decrement	δ	$\delta =\frac{1}{n}{log}_{e}\frac{u_{i}}{u_{i+n}}$	$u_{i}$: amplitude of peak i $u_{i+n}$: amplitude of peak i+n
Viscous damping ratio	ζ	$\zeta =\frac{c}{c_{cr}}$	c: viscous damping coefficient $c_{cr}$: critical viscous damping coefficient
Half-power bandwidth (unit: Hz)	∆ω	$∆\omega =\omega_{2}-\omega_{1}$	$\omega_{1}$: lower half-power frequency $\omega_{2}$: higher half-power frequency
Inverse quality factor	$Q_{inv}$	$Q_{inv}=\frac{1}{Q}=\frac{∆\omega }{\omega_{n}}$	Q: quality factor $\omega_{n}$: natural frequency
Phase lag (unit: radian)	ϕ	$\phi =∠\left(\sigma ,\varepsilon \right)$	$∠\left(\sigma ,\varepsilon \right)$: phase angle between the stress (σ) and strain (ε)
Shear viscosity (unit: Pa·s)	μ	$\mu =\frac{\tau \left(t\right)}{\dot{\gamma }\left(t\right)}$	$\tau \left(t\right)$: shear stress $\dot{\gamma }\left(t\right)$: shear strain rate t: time
Structural reverberation time (unit: s)	$T_{60\;dB}$	$T_{60\;dB}=t_{L-60\;\mathrm{dB}}-t_{L}$	$t_{L-60\;\mathrm{dB}}-t_{L}$: 60 dB decay time
Decay ratio	γ	γ=c/a	a and c: amplitudes of the first and second peaks in step response, respectively

**Table 3:** The important damping parameters measured in practical applications and their relations to other important damping parameters.

Measured Parameter(s)	Target Parameter(s)	
***Dissipated energy*** per cycle (∆W)	***Viscous damping coefficient*** (c): $c=\frac{∆W}{2\pi^{2}B^{2}f}=\frac{∆W}{\pi B^{2}\omega }$	B: displacement amplitude f: frequency in Hz ω: frequency in rad/s
***Dissipated energy*** per cycle (∆W) and ***maximum stored energy*** (W)	***Specific damping capacity*** (ψ): $\psi =\frac{∆W}{W}$	
	***Loss factor*** (η): $\eta =\frac{1}{2\pi }\frac{∆W}{W}$	
***Logarithmic decrement*** (δ)	***Viscous damping ratio*** (ζ): $\zeta =\frac{\delta }{\sqrt{4\pi^{2}+\delta^{2}}}$ $\zeta =\frac{\delta }{2\pi }$ (approx. for small damping, ζ≪1)	
***Loss modulus***$\;(E^{\prime \prime })$ and ***storage modulus*** $\left(E^{\prime }\right)$	***Loss factor*** (η): $\eta =\frac{E^{\prime \prime }}{E^{\prime }}$	
***Half-power bandwidth***$\;(∆\omega =\omega_{2}-\omega_{1}$) ($\omega_{1}$: lower half-power frequency; $\omega_{2}$: higher half-power frequency; $\omega_{n}$: natural frequency; Q: quality factor)	***Viscous damping ratio*** (ζ) and ***inverse quality factor*** ($Q_{inv}$): $\sqrt{1-{2\zeta }^{2}+2\zeta \sqrt{1+\zeta^{2}}}-\sqrt{1-{2\zeta }^{2}-2\zeta \sqrt{1+\zeta^{2}}}=\frac{∆\omega }{\omega_{n}}=\frac{1}{Q}=Q_{inv}$ $\sqrt{1+2\zeta }-\sqrt{1-2\zeta }=\frac{∆\omega }{\omega_{n}}=\frac{1}{Q}=Q_{inv}$ (approx. for small damping, ζ≪1) or $2\zeta =\frac{∆\omega }{\omega_{n}}=\frac{1}{Q}=Q_{inv}$ (approx. for small damping, ζ≪1)	
	***Loss factor*** (η) and ***inverse quality factor*** ($Q_{inv}$): $\sqrt{1+\eta }-\sqrt{1-\eta }=\frac{∆\omega }{\omega_{n}}=\frac{1}{Q}=Q_{inv}$ $\eta =\frac{∆\omega }{\omega_{n}}=\frac{1}{Q}=Q_{inv}$ (approx. for small and medium damping, η<0.3)	
***Phase lag*** (ϕ)	***Viscous damping ratio*** (ζ): $2\zeta \frac{\omega_{n}}{\omega }=tan\left(\phi \right)$ $2\zeta =tan\left(\phi \right)$ (at $\omega =\omega_{n}$)	$\omega_{n}$: natural frequency ω: excitation frequency
	***Loss factor*** (η): $\eta =tan\left(\phi \right)$	
***Loss factor*** (η)	***Viscous damping ratio*** (ζ): $2\zeta \sqrt{1-\zeta^{2}}=\eta$ 2ζ=η (approx. for small damping, ζ≪1)	
	***Inverse quality factor*** ($Q_{inv}$): $Q_{inv}=\frac{1}{Q}=\frac{∆\omega }{\omega_{n}}=\sqrt{1+\eta }-\sqrt{1-\eta }$ $Q_{inv}=\frac{1}{Q}=\frac{∆\omega }{\omega_{n}}=\eta$ (approx. for small and medium damping, η<0.3)	
***Loss modulus***$\;(G^{\prime \prime }$)	***Viscosity*** (μ): $\mu =\frac{G^{\prime \prime }}{\omega }$	ω: frequency in rad/s
***Structural reverberation time***$\;(T_{60\;dB}$)	***Loss factor*** (η): $\eta =\frac{2.2}{f\cdot T_{60\;dB}}=\frac{6{log}_{e}10}{\omega \cdot T_{60\;dB}}$	f: frequency in Hz ω: frequency in rad/s

## Data Availability

The data supporting the findings of this study are available within the article. The scripts used for processing data presented in this study are available on request from the corresponding author.
